# Enhancing fish Underwater Visual Census to move forward assessment of fish assemblages: An application in three Mediterranean Marine Protected Areas

**DOI:** 10.1371/journal.pone.0178511

**Published:** 2017-06-08

**Authors:** Giulia Prato, Pierre Thiriet, Antonio Di Franco, Patrice Francour

**Affiliations:** 1 Université Côte d’Azur, CNRS, FRE 3729 ECOMERS, Parc Valrose, Avenue Valrose, Nice, France; 2 UMR 7208 BOREA—MNHN, CNRS, UPMC, IRD 207, UCN, UA—Muséum National d'Histoire Naturelle, DMPA, UMR BOREA, Paris, France; 3 Muséum National d'Histoire Naturelle, Station Marine de Dinard—CRESCO, Dinard, France; 4 CRIOBE, USR 3278 CNRS-EPHE, University of Perpignan, CNRS, Perpignan, France; 5 Consorzio Interuniversitario per le Scienze del Mare, CoNISMa, Piazzale Flaminio 9, Rome, Italy; University of Hamburg, GERMANY

## Abstract

Monitoring fish assemblages is needed to assess whether Marine Protected Areas (MPAs) are meeting their conservation and fisheries management goals, as it allows one to track the progress of recovery of exploited species and associated communities. Underwater Visual Census techniques (UVC) are used to monitor fish assemblages in MPAs. UVCs should be adapted to fish abundance, body-size and behaviour, which can strongly affect fish detectability. In Mediterranean subtidal habitats, however, UVC strip transects of one surface area (25x5 m^2^) are commonly used to survey the whole fish assemblage, from large shy fish to small crypto-benthic fish. Most high trophic level predators (HTLPs) are large shy fish which rarely swim close to divers and, consequently, their abundance may be under-estimated with commonly used transects. Here, we propose an improvement to traditional transect surveys to better account for differences in behaviour among and within species. First, we compared the effectiveness of combining two transect surface areas (large: 35x20 m^2^; medium: 25x5 m^2^) in quantifying large, shy fish within and outside Mediterranean MPAs. We identified species-specific body-size thresholds defining a smaller and a larger size class better sampled by medium and large transects respectively. Combining large and medium transects provided more accurate biomass and species richness estimates for large, shy species than using medium transects alone. We thus combined the new approach with two other transect surface areas commonly used to survey crypto-benthic (10x1 m^2^) and necto-benthic (25x5 m^2^) species in order to assess how effectively MPAs protection the whole fish assemblage. We verified that MPAs offer significant protection for HTLPs, their response in terms of biomass and density increase in MPAs was always higher in magnitude than other functional groups. Inside MPAs, the contribution of HTLP reached >25% of total fish biomass, against < 2% outside MPAs. Surveys with multiple transect surface areas allow for a more realistic assessment of the structure of the whole fish assemblage and better assessment of potential recovery of HTLPs within reserves of HTLP.

## Introduction

The use of marine protected areas (MPAs) as a tool for fish conservation and fisheries management throughout the world’s oceans has encouraged the development of non-destructive methods to monitor biodiversity and assess the performance of MPAs [1–3]. One of the most extensively documented effects of protection within MPAs is the recovery (in terms of increased density, biomass and body-sizes) of populations usually targeted by fishing. Monitoring programs to assess this recovery, based on underwater visual census (UVC) surveys, have become widespread [2,4–7]. Several UVC techniques have been developed, ranging from transects or fixed points [1,8–10] to obtaining video from SCUBA divers, remotely operated vehicles (ROV) or rotating remote systems [2,3,11]. For shallow areas, UVC with transects and fixed points are often more convenient than video methods. Despite their known biases (*i*.*e*. observer effects, errors in fish body-size and sampling surface-area estimations) [12–14] and constraints (depth and time dive-limits), SCUBA observations are usually cost-effective. Moreover, they enable the detection and identification of a high number of species, as well as quantitative descriptions of the fish assemblage such as density and biomass which are difficult if not impossible to obtain using video methods [3,15,16]. Nonetheless, it is often agreed that UVC methods aiming at quantifying fish abundance through observation within a fixed surface area (i.e. up to a fixed distance from the observer, such as strip transects or fixed points) under-estimate fish density, due to problems in detecting subjects within the sample surface area [17]. Sampling surface areas should be adapted to different fish characteristics: fish density (the sampling surface area must ensure a reasonable probability of encounter: usually, the less abundant is a species, the larger the sampling surface area required), minimum distance of fish approach (behavioural response to diver), species detectability based on size; micro-habitat use, body shape (e.g. flat fish) and color (e.g. sandy gobids) [5,18–20]. According to fish size and behaviour for instance, most fish can be broadly grouped into three categories, and the surface area of a visual census sampling unit should be adapted differently to each [4,19,21]. Moreover, different life stages of the same species might fall into different categories depending on their size (proxy of age and experience, hence determining behavioural response to observer, as well life-stage-specific micro-habitat use). The three main categories are (1) crypto-benthic fish and juveniles of necto-benthic fish spending most of their time hidden within macrophyte stands, holes and crevasses, or resting motionless but camouflaged upon or in the substrate, because of their coloration and/or suitable body-shape (e.g. flat fish). The detectability of these species is low and declines sharply as a function of distance from the observer. They should thus be generally surveyed within small surface areas (0.5–1 meter from the observer) [22,23,24]. (2) Small-medium necto-benthic fish that are generally easy to detect and not strongly affected by diver presence [19] are usually surveyed at a medium distance from the observer (2.5 m from the observer). (3) large fish tend to be shy and to show avoidance behaviour toward the observer [25]. These fish usually occur at lower densities compared to other fish categories, due to (1) the natural lower densities of larger species [26] and (2) they are generally the main fishing targets. Hence, in order to account for the lower detectability of large shy fish at short distances due to shyness, as well as in small sampling surface areas due to lower density, large shy fish need to be surveyed at a greater distance from the observer and within a larger surface area than small-medium necto-benthic fish [19,21]. Distances from the observer adopted in the literature for these fish range from 5 to 15 meters [1,6,17,19].

Although adopting different transect surface areas to survey fish of different size and behaviour is a common practice in monitoring programs carried out in coral reefs [27–29], this is not the case in Mediterranean monitoring programs, even if UVC by strip transects is the most widely used method (Prato et al. in prep and references therein). Variable transect surface areas were used in studies specifically targeting both crypto-benthic (1–2 meters width) and necto-benthic fish (4–5 meters width) [30,31], but a single transect surface area was always used to target both small-medium necto-benthic fish (< 50 cm total length) and larger and shy fish. The most common transect widths adopted in the Mediterranean, moreover, do not exceed 5 meters, meaning a distance of 2.5 meters from the observer [7] (Prato et al in prep. and reference therein), thus possibly inducing underestimation of the abundance of large shy fish that often flee when the observer is approaching.

On the basis of the above-mentioned premises on fish detectability, we hypothesize that standard transects adopted in the Mediterranean (25 x 5 m^2^) are appropriate for adults of small-medium necto-bentic fish and also for the sub-adult stages of “large species”, i.e. species reaching large body lengths at adult age (> 50 cm total length, hereafter referred to as “large shy fish”). Sub-adult stages of these large shy species share comparable total body length and similar behaviour to other small-medium necto-benthic species. However, adult stages of these large shy species are likely to be better sampled in transects of larger surface area. A transect width of 20 meters (i.e. 10 meters on each side of the diver) could encompass a large enough sample area for these species. Similar distances were selected in UVC studies including large shy fish, although in some of those cases fixed points were used [5,17]. In order to obtain density and biomass estimates for these large shy species, transects of different surface areas should be used for different size classes within each species.

Monitoring programs combining different transect surface areas to survey adults of large shy fish, sub-adults of large shy fish and small-medium necto-benthic fish, crypto-benthic fish and juveniles, are therefore urgently needed to be able to more realistically assess the fish assemblage structure and the response to protection of different functional groups. This study has thus two main objectives, schematized in Table 1. Our first objective was to compare the effectiveness of combining two transects surface areas (35 x 20 m^2^ and 25 x 5 m^2^) against only one transect surface area (25 x 5 m^2^) in quantifying the density and biomass of large shy fish inside and outside Mediterranean MPAs. To pursue this objective, we first identified the species-specific fish size threshold discriminating a larger size class of fish that are more accurately sampled with large transects and a smaller size class of fish more accurately sampled with medium transects. Our second objective was to combine the new approach for large shy species with two widely used transect surface areas to survey crypto-benthic fish and juveniles (small transects 10 x 1 m^2^) and necto-benthic fish species (medium transects 25 x 5 m^2^), in order to quantify the effect of protection on the whole fish assemblage and on each functional group, and to quantify the relative contribution of each functional group to total fish biomass.

**Table 1:** Transect length and width used to survey fish groups.

Functional groups	Transect size (width x length in meters) used to survey fish groups			**Biomass, density and effect size
	1 x 10	5 x 25	20 x 35	
	Crypto-benthic fish	Small-Medium necto-benthic fish (max TL < 50 cm)	Large shy necto-benthic fish (max TL > 50 cm)	
Crypto-benthic carnivores	Juveniles and (sub-)adults			**
Juveniles of necto-benthic fish	All but planktonivores (TL≤ 5 cm), Planktonivores (TL ≤ 4 cm)			**
Shoaling planktonivores		(Sub-)Adults (TL > 4 cm)		**
Herbivores		(Sub-)Adults (TL > 5 cm)		**
Necto-benthic carnivores		*(Sub-)Adults (5 cm < TL ≤?) & (? < TL < max TL)	*(Sub-)Adults (5 cm < TL ≤?) & (? < TL < max TL)	**
High trophic level predators		*(Sub-)Adults (5 cm < TL ≤?) & (? < TL < max TL)	*(Sub-)Adults (5 cm < TL ≤?) & (? < TL < max TL)	**

Fish groups in columns are based on micro-habitat use, behavior toward the diver and life stage. TL = total length.

* Cells with one asterisk (*) indicate the first objective of the study: identification of the TL value(“?”) discriminating larger fish better sampled by large transects and smaller fish better sampled by medium transects and assessment of the best combination of transect sizes to survey Large shy necto-benthic fish

** Cells with two asterisks (**) indicate the second objective of the study: assessment of the density and biomass of each functional group (rows) and the evaluation of reserve effect.

## Methods

The study did not involve any experiment or collection of animals. Permission for field surveys was granted by Université Nice Sophia Antipolis.

### Study area

**Fig 1:**
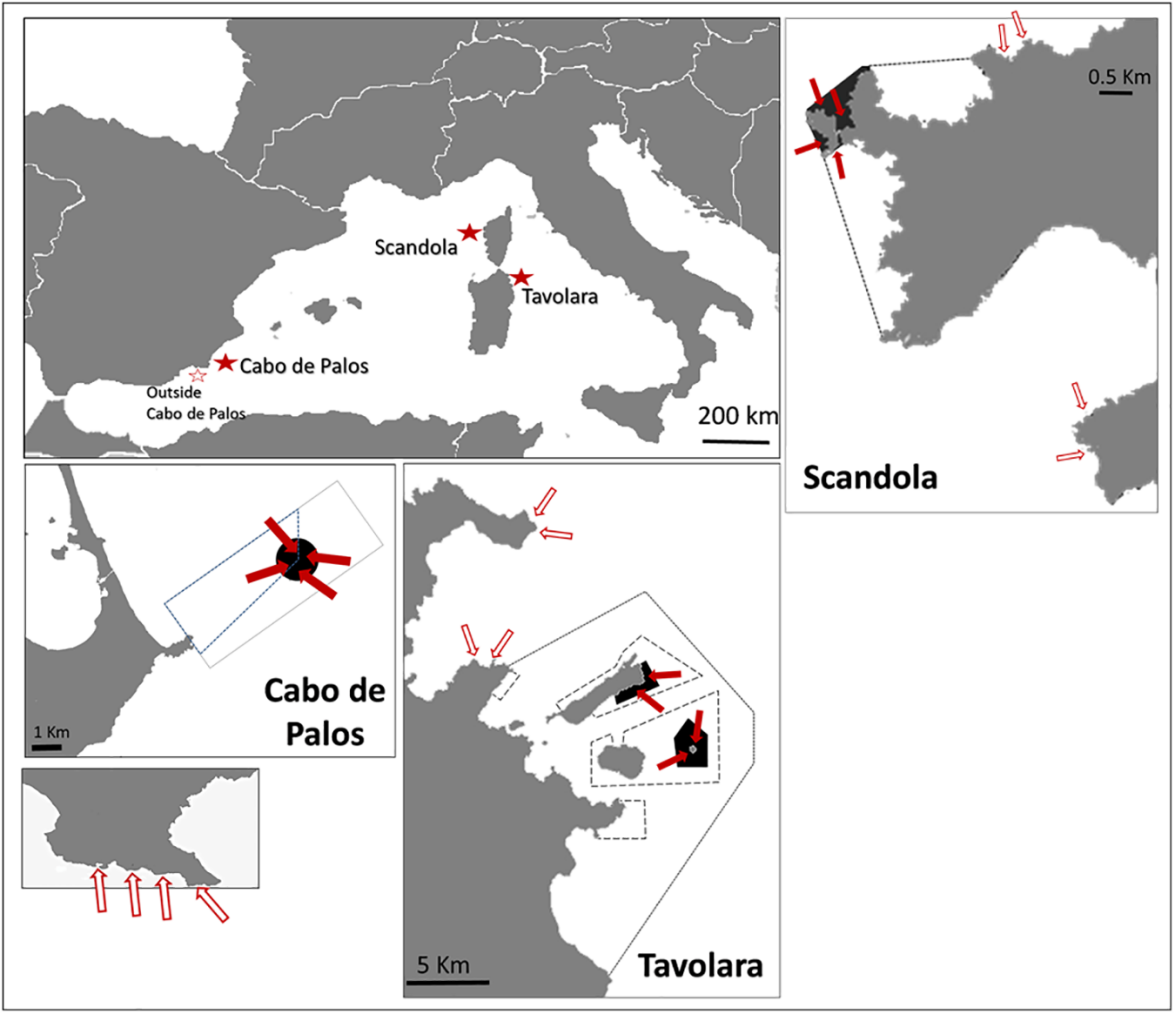
Location of the sampling sites. Sites inside the no-take zones are indicated by filled arrows, sites outside each MPA are indicated by empty arrows. Black filled areas delimit the no-take zones, while dashed borders delimit buffer zones.

This study was performed at three MPAs in the Western Mediterranean (Fig 1). The Tavolara-Punta Coda Cavallo MPA (hereafter Tavolara MPA) is located in north-east Sardinia (Italy, 40° 35’ N, 09° 49’ E) and was established in 1997, but enforcement became effective around 2003–04. It includes 76 km of coastline, covers 15,357 ha, and is separated into three subareas characterised by different levels of protection. In the integral reserve zone (no-take zone, 529 ha), only research activities are allowed, while in the two partial protection zones, different activities are allowed subject to regulations. Spearfishing is not allowed inside the MPA.

The Cabo de Palos-Islas Hormigas MPA (hereafter Cabo de Palos MPA) is located in south-eastern Spain, and was established in 1995. Enforcement has been high in the MPA since its establishment [32]. The MPA extends from the coast to the Islas Hormigas archipelago (37° 38’ N, 0° 42’ W), with a total surface area of 1,898 ha divided into two zones: a no-take zone of 270 ha, surrounding the Hormigas islands archipelago, where only scientific research activities are allowed, and a partial reserve zone where small scale fishing, recreational diving and boating are allowed with some limitations. Recreational fishing, including spearfishing, is not allowed inside the MPA.

The Scandola MPA is located in north-west Corsica (France) and was established in 1975, with regular enforcement [33]. It extends over 25 km of coastline, with a total area of 1,000 ha. It is divided into a no-take zone of 122 ha and a buffer zone where professional fishing is allowed subject to authorization. In the no-take zone, only scientific research activities and boating respecting speed limits are allowed (anchoring is forbidden). Recreational fishing, including spearfishing, is not allowed inside the entire MPA. In the zones outside the three MPAs activities are allowed subject to national laws.

### Sampling design and data collection

Fish assemblage surveys were conducted at each MPA during 3–4 consecutive days in the warm season under optimal visibility conditions, respectively in summer 2013 for the Tavolara and Cabo de Palos MPAs, and in summer 2014 for the Scandola MPA. At each MPA, on rocky habitats between 5 and 15 meters depth, four sites inside the no-take zone and four sites outside the MPA were randomly selected ≥ 500 m apart from each other. Visual census transects of three different surface areas were used in order to account for different fish groups (Table 1, S1 Table) and to allow assessments on the whole fish community, as required by our second objective. The rope used for UVC surveys had 5-m sections coloured alternatively in red and white. This allowed the observer to calibrate their estimate of 10- and 2.5-m widths prior to the start of the survey.

Large transects (35 x 20 m^2^) were used to record only large shy fish (e.g. *Sciaena umbra*, *Epinephelus marginatus*) at an average speed of 700m^2^ / 5min.

Medium transects (25 x 5 m^2^) were used to record all necto-benthic fish (>5 cm total length), i.e. both small-medium necto benthic fish and large shy fish, at an average speed of 125m^2^ / 8 min. Large, shy fish recorded in large transects were also counted in medium transects to allow us to compare the performance of the two transect surface areas and potentially identify a body size threshold at which medium transects were still appropriate, as required to fulfil our first objective (Tab.1). Finally, small transects (10 x1 m^2^) were adopted to survey crypto-benthic fish (e.g. Blenniidae, Gobiidae, Scorpaenidae) and juveniles of necto-benthic fish taxa (<5 cm total length). *Chromis chromis*, *Boops boops* and *Spicara* spp. Juveniles ≤ 4 cm in total length were counted in small transects since smaller individuals are more commonly seen near refuges [34] as opposed to individuals ≥4 cm in total length more commonly seen in the water column, and thus better surveyed with the 25 x 5 m transects (Table 1 and S1 Table).

The number of fish encountered was recorded up to 10 individuals, whereas larger groups were recorded using categories of abundance (i.e. 11–30, 31–50, 51–200, 201–500,500–1000 ind.; see [1]). Fish size (total length, TL) was recorded within 5 cm size classes for large sized fish (species maximum size > 50 cm), 2 cm size classes for other necto-benthic fish species and 1 cm for small crypto-benthic fish. Observers were trained with fish silhouettes of known total length before starting the surveys. Fish wet weight was estimated from size data by means of length–weight relationships from the available literature, selecting coefficients referring to Mediterranean samples whenever possible (www.fishbase.org).

At each site, four replicates of each transect size were randomly located within homogenous rocky habitat: first the large transect was surveyed, followed by the medium transect at approx. 25 m distance. The small transect was surveyed on the way back from the medium transect, while rewinding the transect rope. A new set of transects was then started following the same order at approx. 50 m distance.

### Data analysis

#### Objective 1: Combining large and medium transects for estimating population structure of the 8 large shy species. Development

In order to identify a putative body-size threshold that distinguishes, for each species, a smaller size class better sampled by medium transects and a body-size starting from which larger transects provide better estimates than medium transects, we compared for each species and for each 5 cm size-class the density estimated by large and medium transects. For this purpose, all densities were converted to (decimal) number of fish individuals per square meter. Our statistical unit was the mean density for each transect surface at each sampling site (density was averaged over replicates of one transect surface at one site). Not all 24 sampling sites were used. Instead, we used only the sites where at least one fish individual (for that species and size class) was observed using at least one of the 2 transect surfaces. This allowed exclusion of the non-informative double 0 from the density-estimate comparisons. We then computed the variable “large–medium”, as the difference between large and medium density estimates at each site, and we tested whether the median of the variable “large–medium” was significantly different from 0, using Wilcoxon signed-rank test (the comparison of the mean variable using paired t-test was not applicable due to strong deviance from normality assumptions). A variable “large–medium” having significantly positive median meant that large transects estimated greater densities than medium transects, hence large transects were considered more accurate. A significantly negative median meant the opposite. For each species, this analysis was repeated for every 5 cm-size class. We then investigated whether 2 groups of 5 cm-size classes could be distinguished for each species: a first group composed of the smaller 5 cm-size classes better sampled by medium transects (with negative medians), and a second group composed of the larger 5 cm-size classes better sampled by large transects (with positive medians). For each species, we found a trend indicating the existence of such a size threshold, although there was overall a lack of significance due to the lack of power directly related to the low sample size. Consequently, to increase the power of the size-threshold test procedure, we computed densities of the 2 size-class groups [minimum observed size; threshold] and] threshold; maximum observed size], and then, as above, we tested using Wilcoxon signed rank test whether the first size class group was better sampled by medium transects and the second size class group was better sampled by the large transects. This second analysis had greater power because of the larger sample size (i.e. the number of sites) obtained by using a wider range of size classes allowing fewer double 0 sites where neither medium nor large transects recorded at least one fish.

#### Objective 1: Combining large and medium transects for estimating population structure of the 8 large shy species. Test

For each species, once the threshold was identified, we computed the species density and species biomass by summing up the densities/biomasses of (1) fish belonging to the size-class [minimum observed size; threshold] sampled by medium transects, and (2) fish belonging to the size-class] threshold; maximum observed size] sampled by large transects. These estimates of species density and biomass are hereafter referred to as “Large and medium transects combined” estimates. They are opposed to “Medium transects” estimates, which use density/biomass estimates obtained using only medium transects for all size classes.

In order to assess the gains in using the new method “Large and Medium transects combined” instead of the standard method “Medium transects” when sampling the 8 large shy species, we compared the density and biomass estimates obtained by the two methods at species level and at the level of the 8 species pooled.

At species level, we computed—for species density and species biomass separately—the variable “Large and Medium transects combined–Medium”, as the difference between “Large and Medium transects combined” and “Medium transects” estimates at each site. We then compared the two estimates with Wilcoxon-signed rank test, following the same procedure as described in step 1.

At the level of the 8 species pooled, we computed total density, total biomass and species richness for each of the 2 methods “Large and Medium transects combined” and “Medium transects”, and compared estimates between methods using all sites pooled (24 sites), and separately for each protection level (12 protected sites and 12 unprotected sites). These larger sample sizes allowed use of permutations to assess p-values. We thus used permutational paired t-test (free from normality assumptions because of permutations), more powerful than Wilcoxon signed-rank test.

#### Objective 2: Analyzing the ecological effects of protection on the functional structure of the whole fish assemblage, through the complementary use of “Large and Medium transects combined”, “Medium transects” and “Small transects”. Preliminary data preparation

Every single fish, depending on its taxon and body-size, was assigned to one of the three sampling methods, “Large and Medium transects combined”, “medium transects” or “Small transects”, and to one of the six functional groups based on trophic functions following [35], and on the different micro-habitat uses specific to taxa and/or size class (as a proxy of life stage). Functional groups were: high trophic level predators (HTLP), necto-benthic carnivores, juveniles of necto-benthic fish (which have a crypto-benthic behaviour pattern), crypto-benthic carnivores, shoaling planktonivores and herbivores (S1 Table).

As in the previous analyses, the statistical unit was the site, density variables (at the scale of species, functional groups or the whole fish assemblage) were expressed as (decimal) number of fish per square meter, biomass variables were expressed as grams per square meter and taxa richness was the number of taxa observed per site (by pooling replicates).

#### Objective 2: Analyzing the ecological effects of protection on the functional structure of the whole fish assemblage, through the complementary use of “Large and Medium transects combined”, “Medium transects” and “Small transects”. Analysis 1

In order to assess the magnitude of the effects of protection on the whole fish assemblage, and to compare the magnitude of protection effects among fish at the species and functional group levels, we computed effect sizes (ES) based on Cohen’s index [36] for each of the following variables: total density, total biomass and taxa richness for the whole fish assemblage, total density and total biomass per species, total density and total biomass per functional group.

For each variable, ES was calculated for each of the 3 MPAs as the difference between (1) the average of the variable over the 4 protected sites and (2) the average of the variable over the 4 unprotected sites, divided by the cumulated standard deviation of the variable (*i*.*e*. considering the 8 sites). Then, we computed the mean ES (ES averaged over the MPAs) and its 95% confidence intervals (CI), with positive mean ES indicating positive protection effect, i.e. increased density, biomass, or taxon richness inside MPAs compared to outside. The order of magnitude of the protection effect was assessed following Cohen’s guidelines [36]: no effect: |mean ES| < 0.2; small effect: 0.2 ≤ |mean ES| < 0.5; medium effect: 0.5 ≤ |mean ES| < 0.8; large effect: 0.8 ≤ |mean ES| < 1.3; very large effect: 1.3 ≤ |mean ES|. 95% CI was used to account for uncertainty in the identified order of magnitude.

#### Objective 2: Analyzing the ecological effects of protection on the functional structure of the whole fish assemblage, through the complementary use of “Large and Medium transects combined”, “Medium transects” and “Small transects”. Analysis 2

In order to assess the reserve effect on the trophic structure of the whole fish assemblage, we computed the mean absolute biomass (g/m2) and the mean relative biomass (% of total biomass) for each functional group at each MPA and protection level. The two variables were then averaged over the three MPAs, and compared between protection levels using 95% CI. All analyses and graphical visualizations were performed in R Environment [37] using libraries plyr [38], reshape2 [39], coin [40] and ggplot [41]. All data are provided as Supporting Information (S1 Database)

## Results

### Combining large and medium transects for estimating population structure of the 8 large shy species

#### Identifying species-specific body-size thresholds

**Fig 2:**
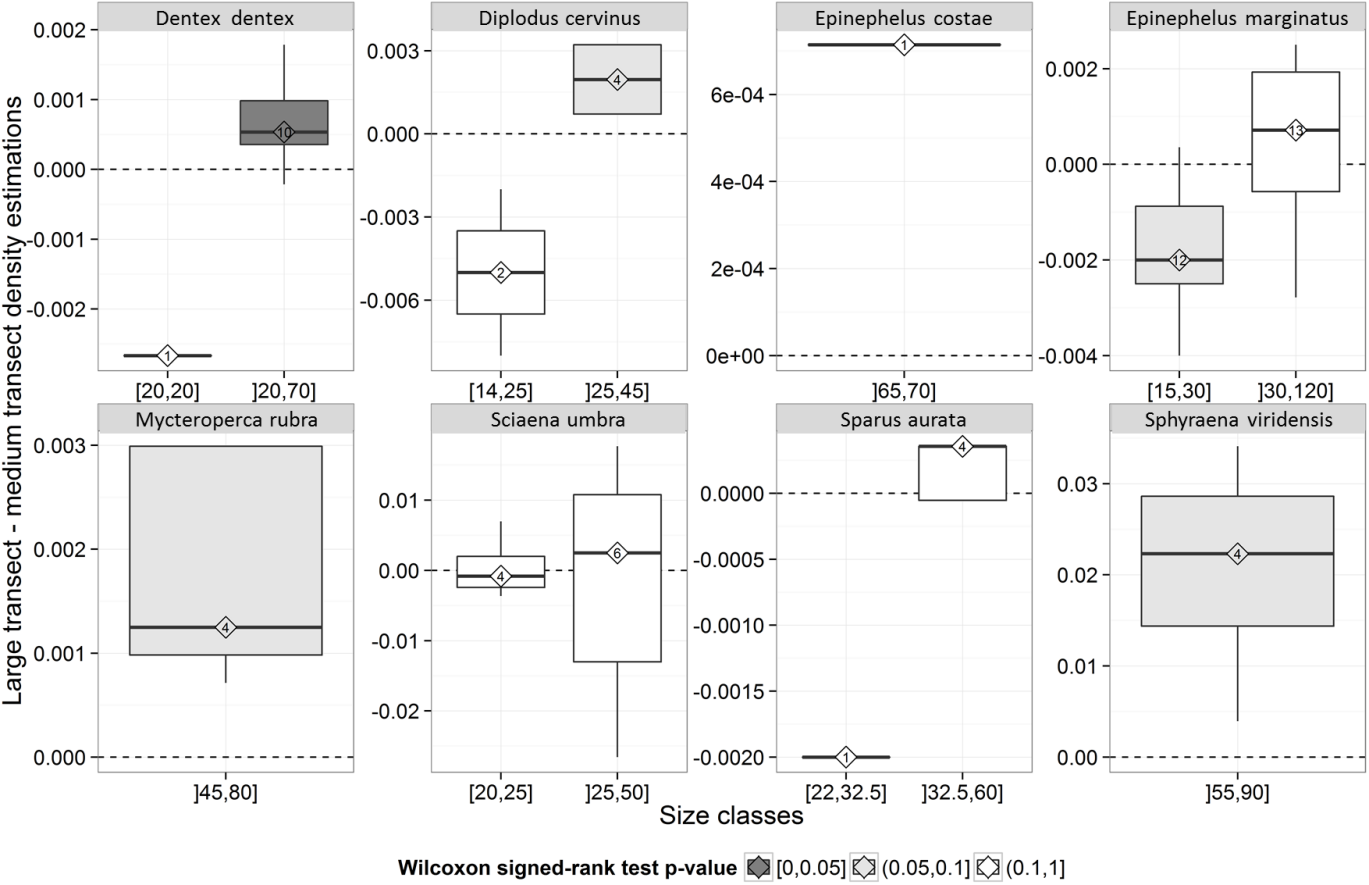
Tukey box plot comparing mean density estimates of large and medium transects. for two size classes of each species. Numbers inside diamonds are the number of sites considered for the comparison (see text for details). The color of the box indicates the significance of the p values based upon Wilcoxon signed rank test. The bottom and top of the box are the first and third quartiles, the band inside the box is the median, the end of the lower whisker is the lowest datum within 1.5 interquartile range (IQR) of the lower quartile, and the end of the upper whisker is the highest datum within 1.5 IQR of the upper quartile

Visual inspection of the difference in density estimates between large and medium transects for each 5 cm size class (S1 Fig) allowed to find species-specific body-size thresholds distinguishing the smaller size class group better sampled by “medium transects” and the larger size class group better sampled by “large transects”. Such thresholds were then tested for significance (Fig 2). Large individuals (above the size threshold) of *D*. *dentex* were detected significantly better by large transects than by medium transects (Fig 2). Trends were also consistent for *M*. *rubra*, *S*. *viridensis*, *D*. *cervinus*, *E*. *costae*, *S*. *umbra*, *E*. *marginatus and S*. *aurata*. Small individuals of *E*. *marginatu*s were significantly better detected by medium transects. For each species, patterns thus consistently showed the higher accuracy of larger transects in sampling the larger size class group, and of medium transects in sampling the smaller size class group. For each species, biomass and density were hence computed by summing biomass and density of the smaller size class group (calculated from medium transects) with biomass and density of the larger size class group (calculated from large transects). S1 Table reports the transect surface area used in the following analysis for abundance estimates of each size class of the 8 large shy species.

#### Effectiveness of the new method “Large and Medium transects combined” VS the standard method “Medium transects”

**Fig 3:**
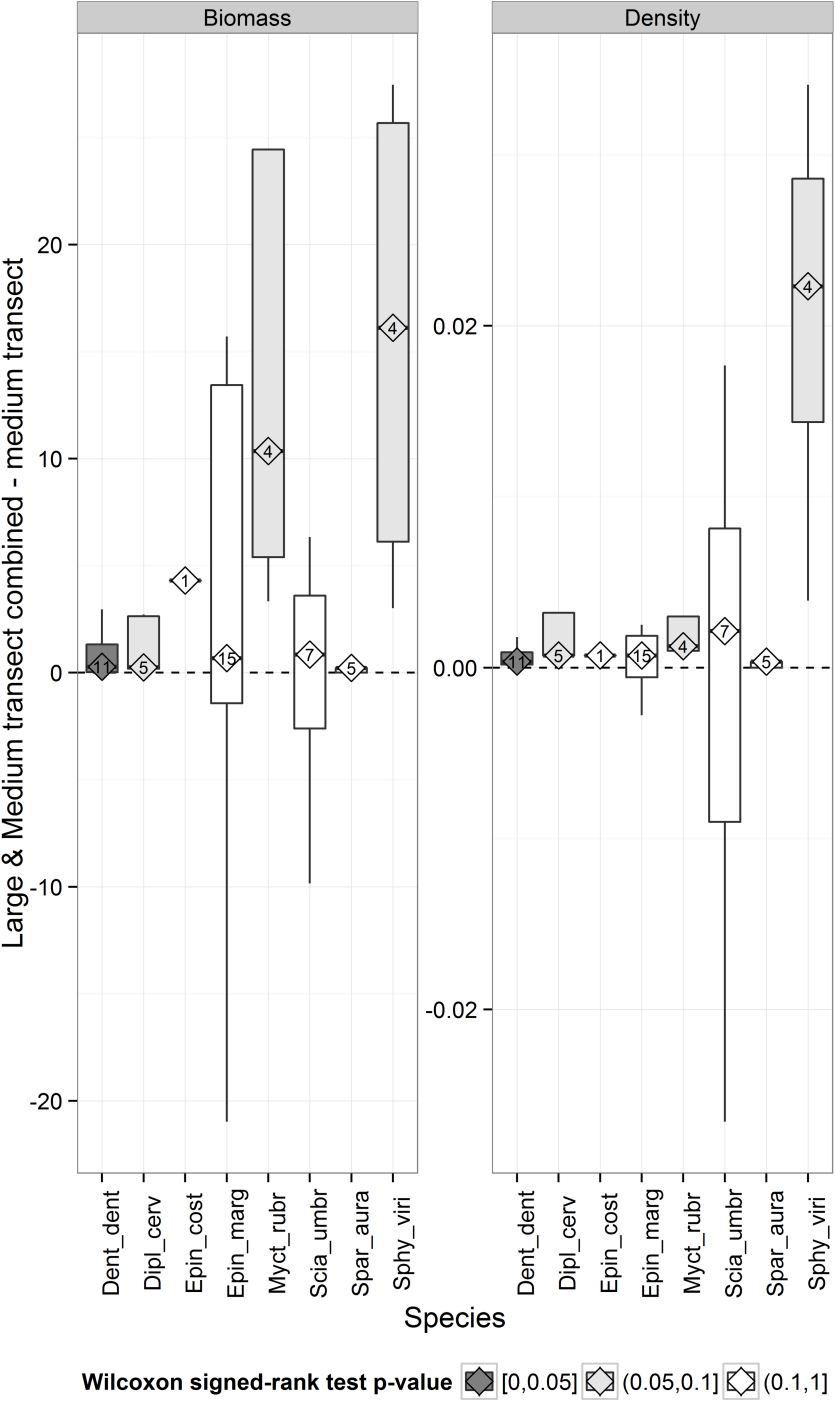
Comparison of mean density and biomass estimated by “Large and Medium transects combined” and by “Medium transects” for each species. Tukey box plots. Numbers inside diamonds are the number of sites considered for the comparison (see text for details). The colour of the box indicates the significance of the p values based upon Wilcoxon signed rank test. The bottom and top of the box are the first and third quartiles, the bend inside the box is the median, the end of the lower whisker is the lowest datum within 1.5 interquartile range (IQR) of the lower quartile, and the end of the upper whisker is the highest datum within 1.5 IQR of the upper quartile.

The new method “Large and Medium transects combined” allowed detection of higher density and biomass estimates for all species (i.e. always positive median values) (Fig 3), although statistical significance was detected only for *D*. *dentex*.

**Fig 4:**
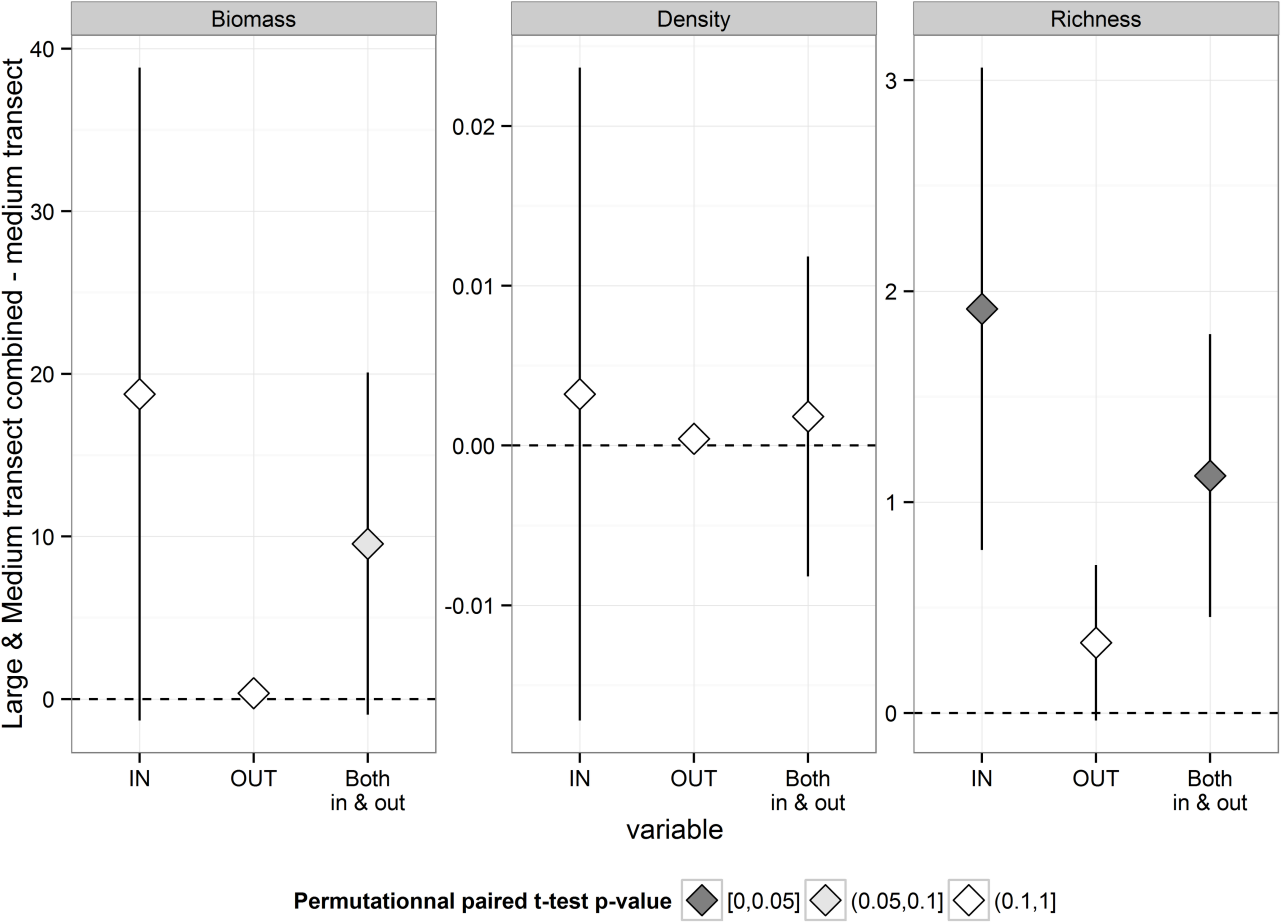
Comparison of mean density, biomass and species richness estimated by “Large and Medium transects combined” and by “Medium transects” for 8 species combined, at each protection level and at all at all sites combined. Bars indicate 95% confidence intervals. The colour of the diamonds indicates the significance of the p values based upon permutational paired t-test.

When the two methods were compared for the 8 species combined and at all sites combined, significantly higher values of species richness were found for the new versus the standard method (Fig 4). However, biomass and density also tended to be higher using the combined method. When the two methods were compared at locations either inside or outside MPAs, few statistical differences were found likely due to the low number of replicates, in particular for outside comparisons. However, trends were always consistent. Outside MPAs, the low abundance of large shy fish in fact caused the occurrence of several “double 0” sites (where no individuals were detected either by large or by medium transects), which reduced the difference between methods.

### Ecological analysis of reserve effects, through the complementary use of “Large and Medium transects combined”, “Medium transects” and “Small transects”

The results obtained from the methods comparison allowed to extend the analysis to the whole fish assemblage through the complementary use of “Large and Medium transects combined”, “Medium transects” and “Small transects”.

**Fig 5:**
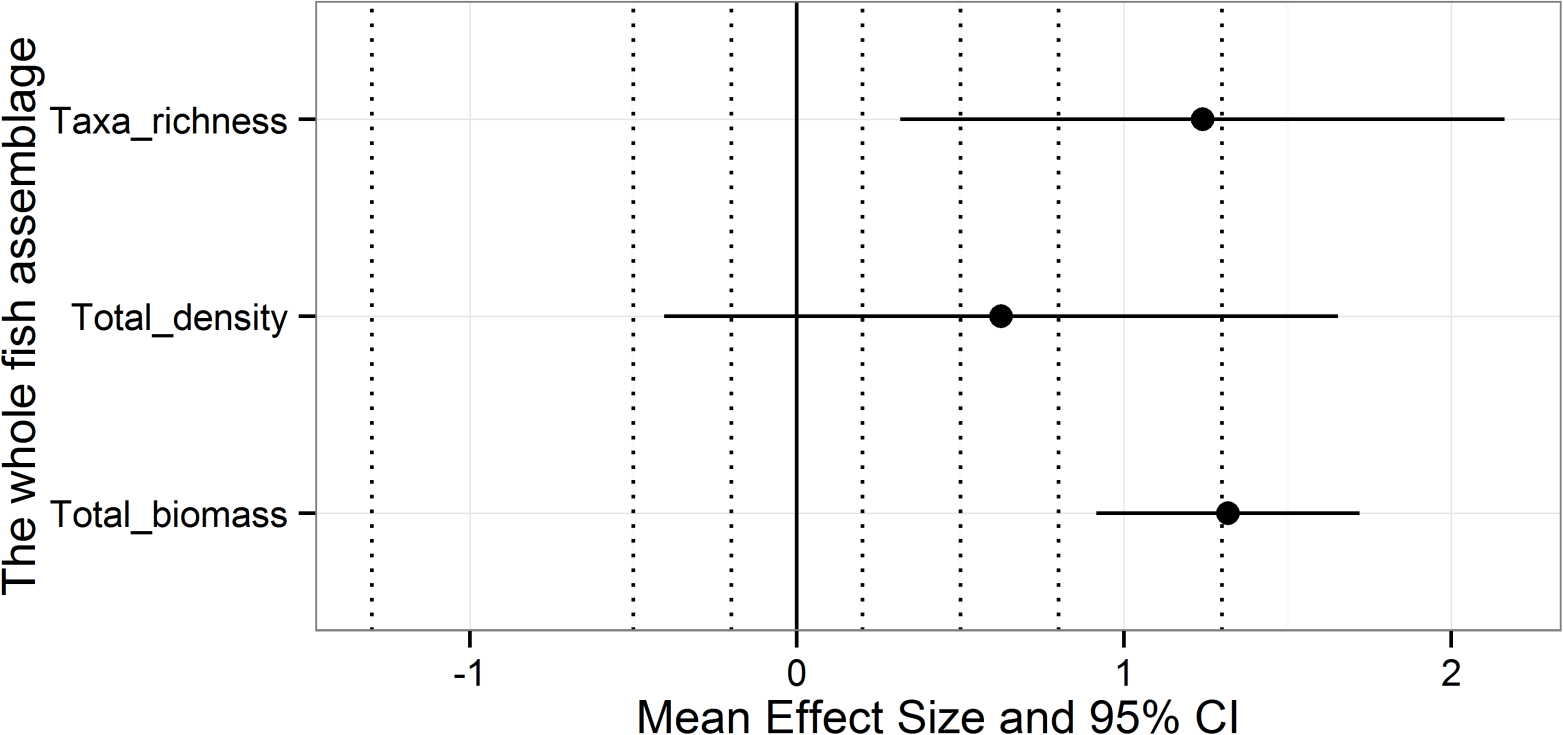
Average effect size ± CI 95% calculated on mean biomass, density and taxa richness for the full fish assemblage. Dashed vertical bars indicate the order of magnitude of the effect size according to Cohen’s guidelines. Starting from 0 and moving both to the positive and negative side of the plot, the bars correspond to: small effect, medium effect, large effect and very large effect.

At the scale of the whole fish assemblage (Fig 5), the effect of protection on taxa richness was positive and large according to Cohen’s guidelines, with CI ranging from small (0.2 < 95% CI inf. limit) to very large (1.3 < 95%CI sup. limit). The protection effect on total density was positive and medium, with CI ranging from negatively medium (-0.2> 95% CI inf. Limit) to positively very large (1.3 < 95% CI sup. limit). The protection effect on total biomass was positive and very large, or at least large (0.8< 95% CI inf. limit).

**Fig 6:**
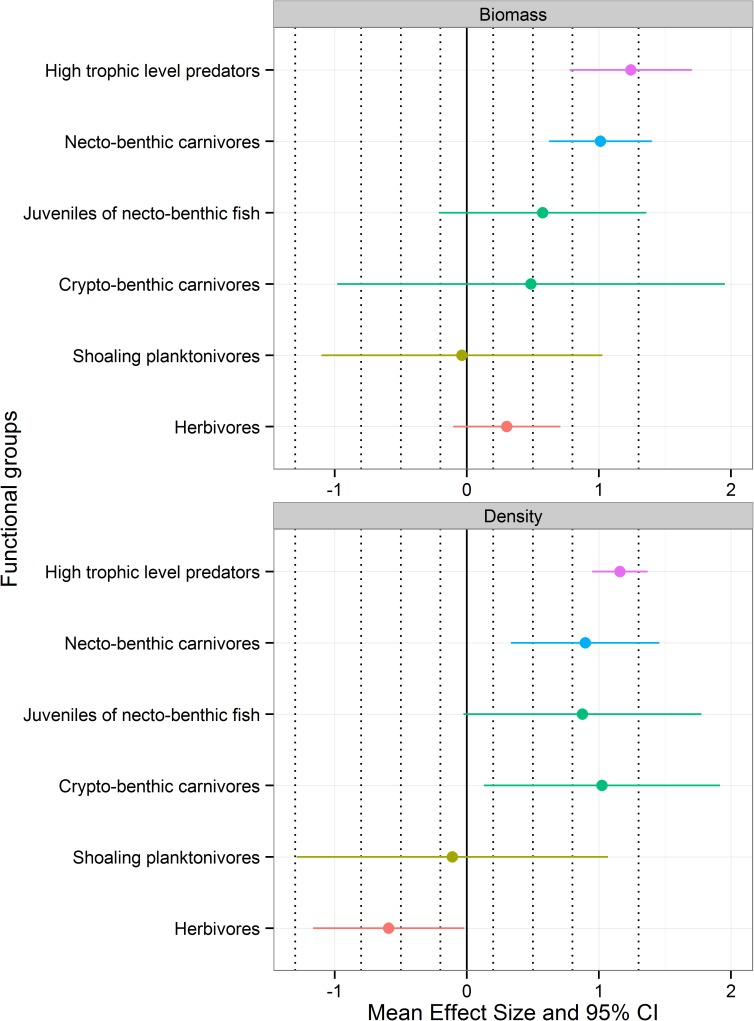
Average effect size ± CI 95% calculated on mean biomass and density for each functional group. Dashed vertical bars indicate the order of magnitude of the effect size according to Cohen’s guidelines. Starting from 0 and moving both to the positive and negative side of the plot, the bars correspond to: small effect, medium effect, large effect and very large effect.

At the scale of functional groups (Fig 6) and for the biomass variable, the effect of protection on HTLP was always positive and large. In order of magnitude of the effect size, HTLP were followed by necto-benthic carnivores, with a large positive ES. Juveniles of necto-benthic taxa and crypto-benthic carnivores showed a positive response to protection too, with medium ES, but larger CI. No protection was indicated for shoaling planktivores. Finally, herbivores showed a small positive effect of MPA presence. When density was analysed, trends were consistent for HTLP, necto-benthic carnivores and shoaling planktivores. Juveniles of necto-benthic fish and crypto-benthic carnivores showed a positive medium effect of protection and a CI which ranged from no effect to a very large effect. Finally, herbivores showed a negatively medium effect of protection.

When ES was computed for each taxon, all species in the HTLP group showed a positive effect of protection both for biomass (S2 Fig) and density (S3 Fig), and among them, *E*. *marginatus* showed the largest positive response to protection (very large ES on density and large ES on biomass). In the other functional groups, response to protection varied among taxa, with both positive and negative effect sizes. *Sarpa salpa* showed an opposite effect to protection when biomass (positive ES) and density (negative ES) were considered.

**Fig 7:**
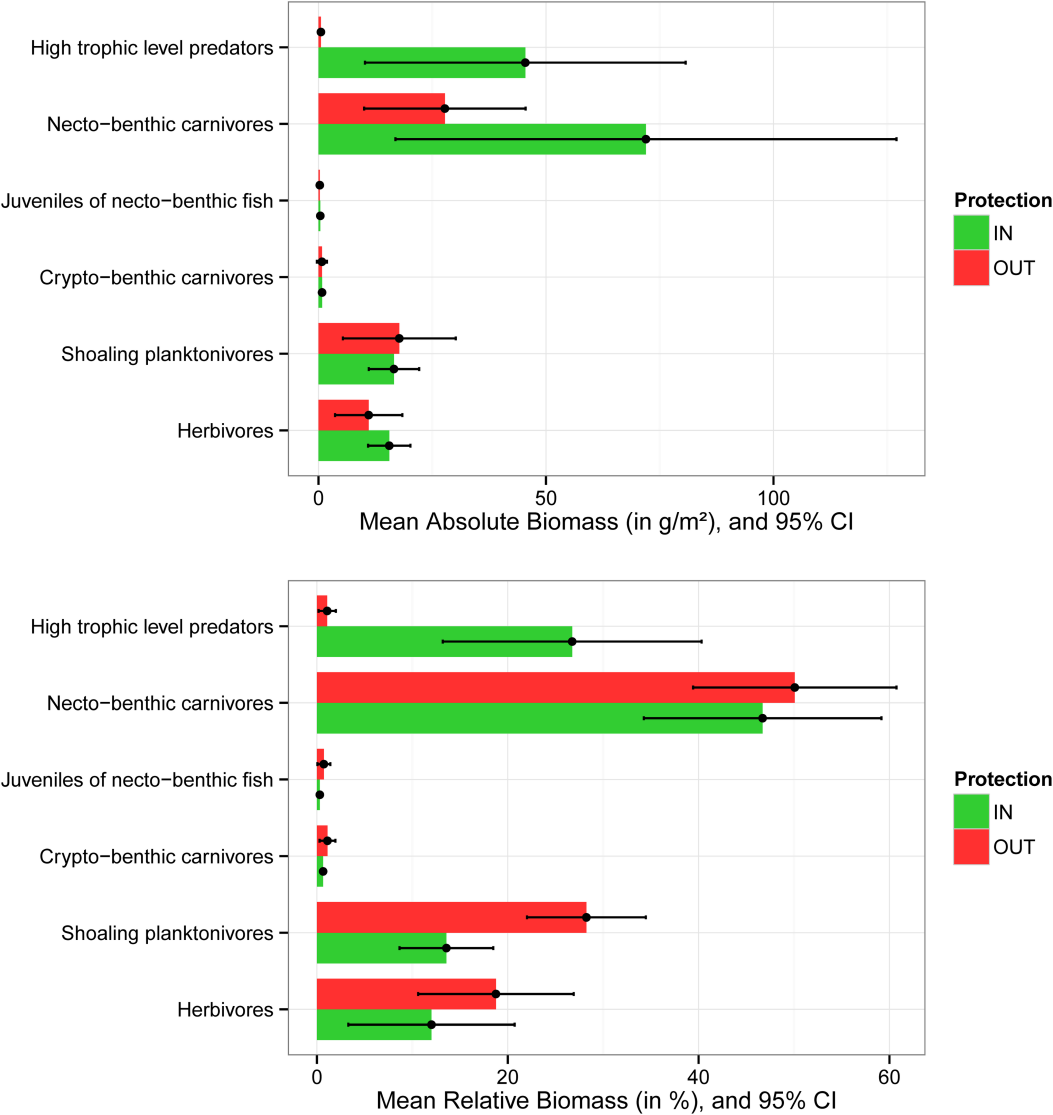
Mean absolute and relative biomass (+- 95% CI) of each functional group inside and outside MPAs.

Analysis of the average contribution of each functional group to total fish biomass revealed the differences among trophic pyramids inside and outside MPAs. Inside MPAs, the contribution of HTLP reached >25% of total fish biomass (45.4 +- 35.2 g/m^2^), against <2% (0.5 +- 0.5 g/m^2^) outside MPAs. Adult necto-benthic fish dominate trophic pyramids both inside (72 +- 55 g/m^2^) and outside MPAs (28 +- 18 g/m), reaching 46% and 50% of total fish biomass respectively. Shoaling planktonivores and herbivores showed a higher contribution to total fish biomass outside MPAs, respectively 28% (17.8 +- 12 g/m^2^) and 18% (11 +- 7.4 g/m^2^) than inside (respectively 12% and 14% of total fish biomass). Similarly, the contribution of crypto-benthic carnivores and juveniles to total fish biomass was higher outside MPAs than inside (Fig 7).

## Discussion

Here, we highlight how combining large (20 m x 35 m) and medium (5 m x 25 m) transects to survey different size classes of large, shy fish increases the accuracy of density and biomass estimates for these species and reduces the potential bias of under-estimation of these species due to diver avoidance. Next, through the adoption of three transect sizes to survey the whole fish assemblage at three Mediterranean MPAs, we detected a significant effect of protection on high trophic level predators, the response of which was always higher in magnitude than that of other functional groups. Trophic pyramids differed inside and outside MPAs due to the larger contribution of HTLP to total fish biomass at protected sites, suggesting this metric as an effective indicator of MPA performance.

### New methodology combining large and medium transects to survey large shy species

Achieving a realistic estimate of fish assemblage density and biomass through visual census is an arduous task. On one hand, many authors agree that UVC underestimates the true abundance of fish, since a human observer will probably always miss a small percentage of fish that are in fact present within the census area (e.g. [42–44]). Thus, when several visual census methods are compared, greater accuracy is generally assumed to be represented by the highest density of fish recorded [5,20]. On the other hand, other authors suggest that underwater visual census may overestimate the abundance of fish because of non-instantaneous counts being performed [45,46]. This bias can occur especially in presence of predator fishes displaying high swimming speed and/or attractive behaviour towards divers [45]. The behaviour of fish is likely to have the greatest effect on the accuracy of the visual census method: simultaneously counting a range of species with different behaviour leads to lower accuracy in estimates than if species groups, and size classes within a species group, are counted separately, using the method best suited to their behaviour [30,47].

Size can indeed be considered a proxy of behaviour: large fish are older and hence, in fished areas, may tend to avoid the diver due to their life experience (i.e. this is not necessarily the case in highly enforced MPAs [48]). In this perspective, we analysed the effectiveness of combining large sized transects and medium sized transects to record large shy fish, that in the Mediterranean are generally surveyed using the same transect dimensions adopted for all necto-benthic fish (Prato et al. in prep). First, we showed that different size classes within a large shy species are better detected with different transect surface areas (larger size classes with larger transects), and we identified species-specific size thresholds. Then, we used these size thresholds to select data from the most appropriate transect surface area for each large shy fish species. Finally, we demonstrated that surveys combining large and medium transects to survey large shy fish, provided more accurate (i.e. higher) abundance and biomass estimates for these species than surveys using only medium transects. In particular, *Dentex dentex*, were always more accurately surveyed with large and medium transects combined (i.e. higher abundances and biomass detected). The same trend was also valid for all other large shy species (median values were positive for *Epinephelus marginatus*, *Sciaena umbra*, *Sparus aurata Diplodus cervinus*, *Epinephelus costae*, *Sphyraena viridensis* and *Mycteroperca rubra*). The lack of statistical difference for these species was probably not related to the absence of a transect size effect, but to the low number of replicates available for this analysis, too few to account for the low abundance of these species at some sites (e.g. *E*. *costae* was detected at only one site). This was confirmed by the comparison between the effectiveness of the two methods at estimating total density, biomass and species richness of the 8 large shy species combined inside and outside MPAs. When all sites were considered irrespectively of the protection factor, the new method allowed detection of significantly higher species richness and a consistent trend of higher biomass than the standard method. When fewer sites, hence fewer replicates, were used for the analysis, statistical differences between the two methods declined, especially outside MPAs. Inside MPAs, the high abundance of large shy species allowed detection of significantly higher species richness and a clear trend of higher species biomass with the new method, despite the low number of replicates. Outside MPAs, in contrast, the low number of transects where at least one individual was detected (due to the low abundance of the 8 species) did not enable enable any statistical difference to be highlighted) between the two transect sizes, although the trend was always consistent.

These results well agree with what was observed by [19], i.e. that larger numbers of large mobile and/or shy fish would be observed further away from the observer than directly on the transect path. Even where these species are abundant, such as in our above-mentioned MPA locations, they will still keep a “safe distance” from the observer, thus the probability of detection (sensu [19]) is higher within a distance of 10 m compared to 2.5 m on each side of the observer. Combining large transect size with medium transect size could thus help to reduce the bias of under-estimation due to fish behaviour. In addition, the possible overestimation bias of non-instantaneous visual counts due to the higher speed of the large fish with respect to the speed of the observer [45] is unlikely to occur here. As also stated in [49], the large shy fish in the analysed ecosystem are not particularly fast-swimming species. Moreover, larger transects were surveyed at an average speed (700m^2^ / 5min) approximately 8 times higher than the speed of survey on medium transects (125m^2^ / 8min), thus overestimation bias due to fish flux across the sampling area is probably reduced. Nonetheless, further testing would be needed to formally address this issue.

Medium transect size appears to be best suited to survey smaller size classes of these large, shy species. For instance, smaller dusky grouper often hide in crevices and thus tend to be overlooked by an observer surveying a larger surface area [18,21]. In medium transects, a smaller area can be more easily searched and, thus, the detection of more sedentary and hidden species is more probable [20]. Although large, shy fish were not considered in their study, [20] compared nested cylinders of varying radius length to survey fish of different size classes, showing that best abundance estimates for small-sized individuals were obtained on smaller surfaces, while larger individuals were recorded with higher accuracy and precision in larger surface area units.

### Ecological analysis of reserve effects, through the complementary use of “Large and Medium transects combined”, “Medium transects” and “Small transects”

Due to the better estimates of biomass and species richness obtained from large and medium transects, we recommend a combination of large, medium and small transect sizes be used to analyse potential impacts of MPAs on the whole fish assemblage. This approach will be particularly important when attempting to assess the relative contribution of high trophic level predators to fish biomass as demonstrated within our three MPAs.

The average response of HTLP to protection was always positive and was higher in magnitude than the response of other functional groups. Necto-benthic carnivores were also always favoured by protection since they included species usually targeted by fishing. Lower trophic level species (including mostly non-commercial species) showed high variability in response, including decline of some species in the MPAs, which highlighted the occurrence of possible indirect negative effects of protection through predation or competition for resources. These results were similar to those of meta-analytical studies encompassing several Mediterranean MPAs [50,51] and corresponded well to the response observed by the same trophic groups in well-enforced MPAs [35]. Nonetheless, although cascading trophic interactions are probably occurring at the species level, at the trophic group level the increase in the biomass of predator groups did not lead to a significant reduction in the biomass of lower trophic level groups inside the MPAs compared to the exploited sites, as could be expected on the basis of increased predation inside the MPA. Exploitation at the non-protected sites is thus likely to have larger impact across the food-web than the top-down control exerted by high trophic level predators at protected sites [52].

Finally, analysis of effect size and of trophic pyramids showed that the biomass of high trophic level predators was the metric that differed most between transects within and outside MPAs. When all functional groups are compared, the biomass of high trophic level predators contributed most to the total fish biomass within MPAs. As also suggested by [52], such disparity in biomass ratios between MPAs and open access sites for the different trophic groups implies a trophic re-organisation that is likely to have substantial consequences for ecological functions Similar trends were observed in a study covering 13 MPAs and 17 unprotected sites across the Mediterranean [49], suggesting that this metric is a useful indicator of MPA performance.

## Conclusion

Previous studies have demonstrated how the abundance of high trophic level predators can increase within MPAs up to several years after the introduction of protection [53], and long-term monitoring programs are, thus, essential to establish when carrying capacity is reached [53]. On the basis of our results, we suggest that monitoring programs adopt transects of variable surface area to best census fish of different size and behaviour, as has long been suggested [1], but never tested in the Mediterranean Sea. The sampling and analysis framework adopted in this study could markedly improve surveys of fish assemblages. Using the combination of large and medium transects to survey large shy fish, allows, with few analytical steps, improvement of the accuracy of estimates for these species, the detection of which is usually hampered by their behavioural traits. The steps include:1-identification of the subset of large shy species that, because of their size and behaviour when adults, are likely to be underestimated in medium transects of 25x5 m (common in the Mediterranean); 2-sampling of this subset of large shy species with both large and medium transects, sampling of other necto-benthic species with medium transect and of cryptobenthic species and juveniles of necto-benthic species with small transects;– 3 -selection of the size threshold for each large shy species, defining the best correspondence between each size class and transect size; 4- -analysis of the full fish assemblage using best estimates of large shy species obtained from large and medium transects combined, estimates of necto-benthic fish from medium transects and estimates of cryptobenthic fish and juveniles from small transects.

In a monitoring program, costs and benefits of each potential methodology must be considered. The approach we propose implies higher costs than standard surveys with one transect size because of the greater sampling effort needed. However, this approach will provide more accurate estimate of the abundance and biomass of large, shy fish (as proven by our analysis)—a category including high trophic level predators- and of cryptobenthic fish and juveniles (as proven by the literature), and thus provides a more realistic description of fish assemblage composition and trophic pyramids. For low-cost assessment of the reserve effect of an MPA (i.e. routine monitoring activity), one transect size is sufficient, However, in the perspective of long-term monitoring programs to assess the recovery of HTLP, a key ecological and commercial group, and the patterns of change in fish assemblage composition in MPAs, the approach we propose should be preferred, since it provides data that would allow a better understanding of ecosystem functioning.

## Supporting information

S1 Table List of surveyed fish species according to transect length and width used and species-specific size thresholds of large shy fish. (DOCX)

S1 Fig Comparison of the density estimated by large and medium transects for each 5 cm- size class of each species. Tukey boxplots. Numbers inside diamonds are the number of sites considered for the comparison (see text for details). The color of the box indicates the significance of the differences in mean density estimates based upon Wilcoxon signed rank test. The bottom and top of the box are the first and third quartiles, the diamond is the median, the end of the lower whisker is the lowest datum within 1.5 interquartile range (IQR) of the lower quartile, and the end of the upper whisker is the highest datum within 1.5 IQR of the upper quartile
(PNG)

S2 Fig Average effect size ± CI 95% calculated on mean biomass for each taxon (PNG)

S3 Fig Average effect size ± CI 95% calculated on mean density for each taxon. (PNG)

S1 Database Database of transect data. (XLSX)
