# Supplementary material for: Enhancing fish Underwater Visual Census to move forward assessment of fish assemblages: An application in three Mediterranean Marine Protected Areas
Source: PLoS One. 2017 Jun 8;12(6):e0178511. doi: 10.1371/journal.pone.0178511 (PMC5464568; doi:10.1371/journal.pone.0178511)
Supplement: S1 Table — (DOCX) [file pone.0178511.s001.docx]

| **Functional groups** | **Transect length and width (m) used to survey fish groups based on micro-habitat use & behavior toward diver .** | | | **Max. TL**  **(cm)** |
| --- | --- | --- | --- | --- |
|  |  |  |  |  |
|  | **1 x 10** | **5 x 25** | **20 x 35** |  |
|  | **Crypto-benthic fish** | **Small-Medium necto-benthic fish** | **Large shy necto-benthic fish** |  |
| Crypto-benthic carnivores | *Scorpaena* spp. |  |  |  |
|  | *Scorpaena scrofa* |  |  | 50 |
|  | *Parablennius gattorugine* |  |  | 16 |
|  | *Parablennius pilicornis* |  |  | 13 |
|  | *Parablennius rouxi* |  |  | 8 |
|  | *Parablennius tentacularis* |  |  | 15 |
|  | *Parablennius zvonimiri* |  |  | 7 |
|  | *Tripterygion delaisi* |  |  | 9 |
|  | *Tripterygion tripteronotus* |  |  | 8 |
| Shoaling planktonivores | *Juv. Anthias anthias* | *Anthias anthias* |  | 25 |
|  | *Juv. Apogon imberbis* | *Apogon imberbis* |  | 15 |
|  | *Juv. Boops boops* | *Boops boops* |  | 30 |
|  | *Juv. Chromis chromis* | *Chromis chromis* |  | 25 |
|  | *Juv. Oblada melanura* | *Oblada melanura* |  | 35 |
|  | *Juv. Spicara spp.* | *Spicara* spp. |  | 25 |
| Herbivores | *Juv. Sarpa salpa* | *Sarpa salpa* |  | 50 |
| Necto-benthic carnivores | *Juv. Coris julis* | *Coris julis* |  | 30 |
|  | *Juv. Diplodus annularis* | *Diplodus annularis* |  | 24 |
|  | *Juv. Diplodus puntazzo* | *Diplodus puntazzo* |  | 50 |
|  | *Juv. Diplodus sargus sargus* | *Diplodus sargus sargus* |  | 45 |
|  | *Juv. Diplodus vulgaris* | *Diplodus vulgaris* |  | 45 |
|  | *Juv. Labrus merula* | *Labrus merula* |  | 45 |
|  | *Juv. Labrus viridis* | *Labrus viridis* |  | 47 |
|  | *Juv. Mullus surmuletus* | *Mullus surmuletus* |  | 40 |
|  | *Juv. Serranus cabrilla* | *Serranus cabrilla* |  | 40 |
|  | *Juv. Serranus scriba* | *Serranus scriba* |  | 35 |
|  | *Juv. Spondyliosoma cantharus* | *Spondyliosoma cantharus* |  | 60 |
|  | *Juv. Symphodus cinereus* | *Symphodus cinereus* |  | 15 |
|  | *Juv. Symphodus doderleini* | *Symphodus doderleini* |  | 10 |
|  | *Juv. Symphodus mediterraneus* | *Symphodus mediterraneus* |  | 18 |
|  | *Juv. Symphodus melanocercus* | *Symphodus melanocercus* |  | 14 |
|  | *Juv. Symphodus ocellatus* | *Symphodus ocellatus* |  | 12 |
|  | *Juv. Symphodus roissali* | *Symphodus roissali* |  | 17 |
|  | *Juv. Symphodus rostratus* | *Symphodus rostratus* |  | 13 |
|  | *Juv. Symphodus tinca* | *Symphodus tinca* |  | 44 |
|  | *Juv. Thalassoma pavo* | *Thalassoma pavo* |  | 25 |
|  | *Juv. Sciaena umbra* | *Sciaena umbra (5 < TL ≤ 25)* | *Sciaena umbra (TL>25)* | 70 |
|  | *Juv. Diplodus cervinus cervinus* | *Diplodus cervinus (5 < TL ≤ 25)* | *Diplodus cervinus (TL>25)* | 55 |
|  | *Juv. Sparus aurata* | *Sparus aurata (5 < TL ≤ 32.5)* | *Sparus aurata (TL> 32.5)* | 70 |
| High trophic level predators | *Juv. Muraena helena* | *Muraena helena* |  | 150 |
|  | *Juv. Dentex dentex* | *Dentex dentex (5 < TL ≤ 20)* | *Dentex dentex (TL>20)* | 100 |
|  | *Juv. Epinephelus costae* | *Epinephelus costae (5 < TL ≤ 65)* | *Epinephelus costae (TL > 65)* | 140 |
|  | *Juv. Epinephelus marginatus* | *Epinephelus marginatus*  *(5 < TL ≤ 30)* | *Epinephelus marginatus (TL>30)* | 150 |
|  | *Juv. Mycteroperca rubra* | *Mycteroperca rubra (5 < TL ≤ 45)* | *Mycteroperca rubra (TL> 45)* | 145 |
|  | *Juv. Sphyraena viridensis* | *Sphyraena viridensis (5 < TL ≤ 20)* | *Sphyraena viridensis (TL>20)* | 130 |

**S1 Table. Transect length and width used to survey fish groups.**

Groups (columns) are based on micro-habitat use, behavior toward the diver and life stage. Rows indicate functional groups based on trophic functions following [41], and on the different micro-habitat uses specific to taxa and/or size class (as a proxy of life stage). TL = total length. Juv = juveniles. Juveniles of planktonivores measure <4 cm TL, juveniles of all other species measure <5cm TL. Juveniles were considered as a separate functional group in data analysis, but are not listed separately in the table for sake of simplicity. TL thresholds (in parenthesis) distinguishing size classes of large shy fish better sampled by large and medium transects were identified in the first objective of the study.
