# Supplementary figures and images for: Enhancing fish Underwater Visual Census to move forward assessment of fish assemblages: An application in three Mediterranean Marine Protected Areas
Source: PLoS One. 2017 Jun 8;12(6):e0178511. doi: 10.1371/journal.pone.0178511 (PMC5464568; doi:10.1371/journal.pone.0178511)

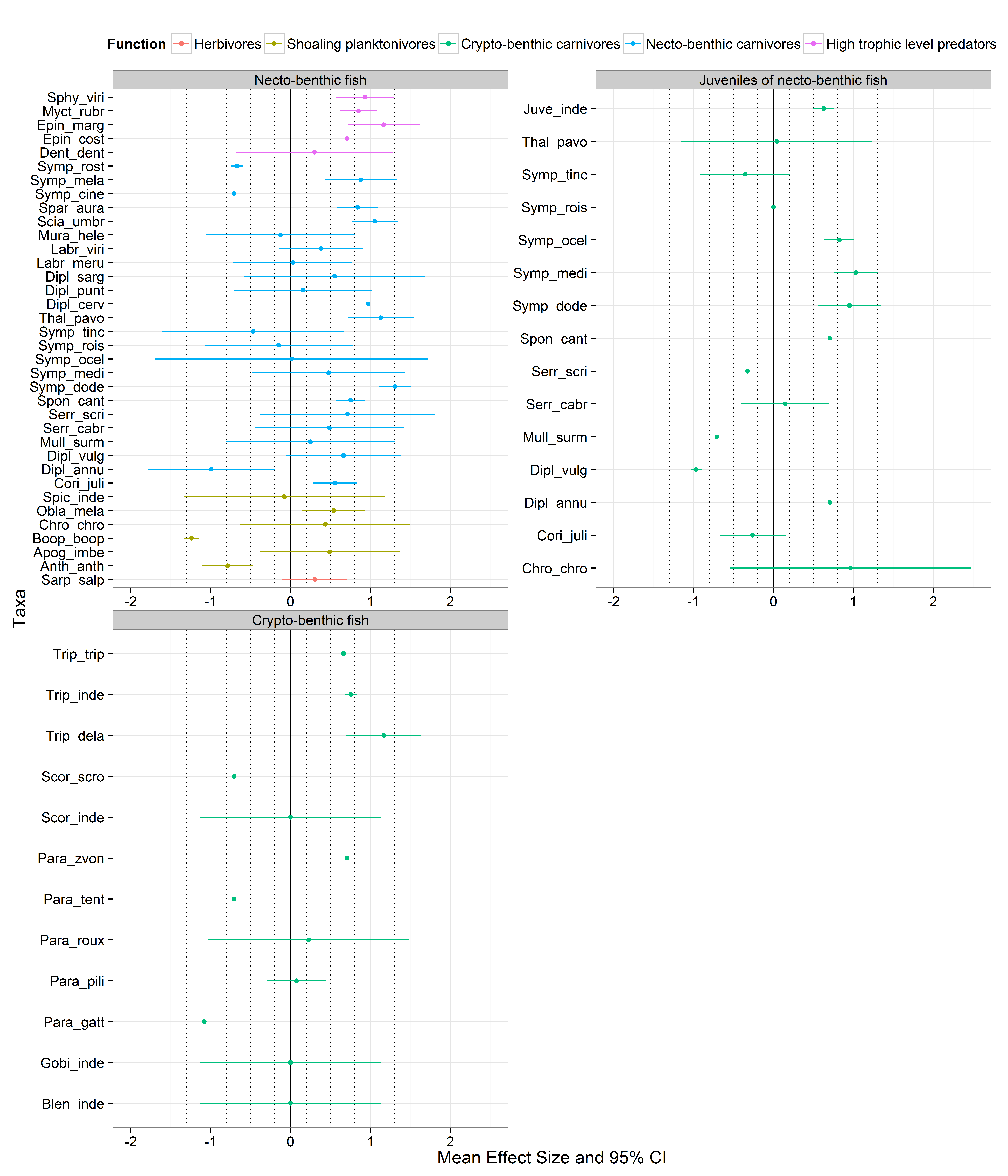

Supplement: S2 Fig — (PNG) [file pone.0178511.s003.png]

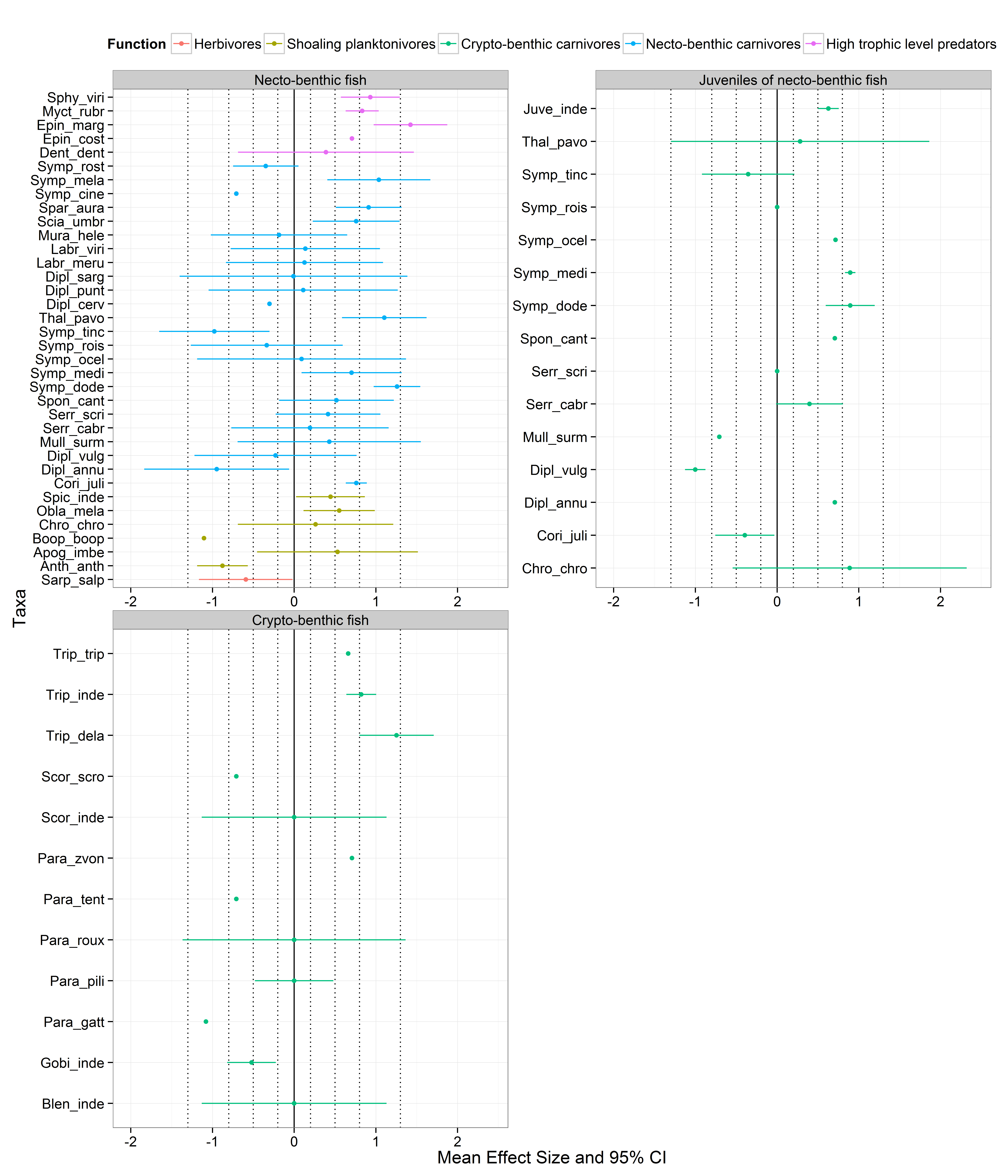

Supplement: S3 Fig — (PNG) [file pone.0178511.s004.png]
